# hCG-induced Sprouty2 mediates amphiregulin-stimulated COX-2/PGE2 up-regulation in human granulosa cells: a potential mechanism for the OHSS

**DOI:** 10.1038/srep31675

**Published:** 2016-08-19

**Authors:** Jung-Chien Cheng, Lanlan Fang, Hsun-Ming Chang, Ying-Pu Sun, Peter C. K. Leung

**Affiliations:** 1 Department of Obstetrics and Gynaecology, Child & Family Research Institute, University of British Columbia, Vancouver, British Columbia, V5Z 4H4, Canada; 2 Reproductive Medical Center, The First Affiliated Hospital of Zhengzhou University, Zhengzhou, 450052, China

## Abstract

Sprouty2 (SPRY2) is an important intracellular regulator for epidermal growth factor receptor (EGFR)-mediated ERK1/2 signaling. In human granulosa cells, although SPRY2 is expressed, its regulation and function remains complete unknown and must be defined. Our previous study has shown that human chorionic gonadotropin (hCG)/luteinizing hormone (LH) up-regulates the expression levels of EGF-like growth factor, amphiregulin (AREG), which subsequently contributes to the hCG/LH-induced COX-2 expression and PGE2 production. The aim of the present study was to investigate the effect of hCG on SPRY2 expression and the role of hCG-induced SPRY2 in AREG-stimulated COX-2 expression and PGE2 production in human granulosa cells. Our results demonstrated that the expression of SPRY2 was up-regulated by hCG treatment. Using pharmacological inhibitors and siRNA knockdown, we showed that activation of ERK1/2 signaling was required for hCG-induced up-regulation of SPRY2 expression. Further, SPRY2 knockdown attenuated the AREG-induced COX-2 expression and PGE2 production by inhibiting AREG-activated ERK1/2 signaling. Interestingly, we showed that SPRY2 expression levels were significantly increased in granulosa cells of ovarian hyperstimulation syndrome (OHSS) patients. These results for the first time elucidate the physiological roles of SPRY2 in human granulosa cells and suggest that aberrant expression of SPRY2 may contribute to the pathogenesis of OHSS.

Sprouty (SPRY) was first identified in *Drosophila*, where it acts as an antagonistic regulator of tracheal branching^1^. Thus far, four SPRY genes (SPRY1-4) have been identified in mammals^2^. SPRY1, SPRY2 and SPRY4 are expressed in various mouse embryonic tissues, whereas SPRY3 is only detected in the adult brain and testis^2^,^3^. Similar to *Drosophila* SPRY, mammalian SPRY proteins can negatively regulate receptor tyrosine kinase (RTK)-mediated signaling by inhibiting the RAS/ERK1/2 signaling pathway^4^,^5^. In bovine granulosa cells, the expression levels of SPRY2 mRNA have been shown to be associated with the oocyte developmental competence^6^. In humans, SPRY2 mRNA and protein have been detected in granulosa cells obtained from patients undergoing *in vitro* fertilization (IVF)^7^. Moreover, the mRNA levels of SPRY2 in the granulosa cells of ovarian follicles obtained from IVF patients who have conceived are significantly higher than those in the granulosa cells of patients who have arrested embryos^8^. These studies suggest that SPRY2 may play important roles in the regulation of follicle development, particularly for the final stage of follicle maturation and corpus luteum formation.

In mammals, ovulation is a tightly regulated process involving the regulation of various gene expression levels, initiated by the surge in luteinizing hormone (LH)^9^. It has been well established that cyclooxygenase-2 (COX-2) and its major product prostaglandin E2 (PGE2) act as critical mediators that regulate ovulation^10^. Epidermal growth factor receptor (EGFR) and its ligands are expressed in reproductive tissues and have been shown to mediate various female reproductive functions^11^. In mouse follicles, treatment with EGF-like growth factors, specifically amphiregulin (AREG), betacellulin (BTC) or epiregulin (EREG), triggers cumulus expansion, oocyte maturation and COX-2 expression^12^. In addition, animal studies demonstrate that inhibition of EGFR tyrosine kinase activity decreases LH- or human chorionic gonadotropin (hCG)-induced ovulation^13^,^14^. Notably, our previous study shows that LH up-regulates the expression levels of AREG, BTC and EREG, which subsequently contribute to the LH-induced COX-2 expression and PGE2 production in human granulosa cells^15^. Taken together, these studies indicate that LH and LH-induced EGF-like growth factors play critical roles in the regulation of ovulation.

It has been shown that LH/hCG treatment significantly up-regulates SPRY2 expression in bovine granulosa cells^16^. However, whether the same is true in human granulosa cells remains unknown. Further, if LH/hCG treatment does up-regulate SPRY2 expression, whether SPRY2 mediates EGF-like growth factor-induced COX-2 expression and PGE2 production in human granulosa cells also remains unknown. In the current study, our results showed that treatment with hCG up-regulated SPRY2 expression in human granulosa cells through activation of the ERK1/2 signaling pathway. In addition, knockdown of SPRY2 attenuated AREG-induced COX-2 expression and PGE2 production. Moreover, we found that the expression levels of SPRY2 were significantly higher in the granulosa cells of ovarian follicles from IVF patients who later developed ovarian hyperstimulation syndrome (OHSS).

## Results

### Treatment with hCG up-regulates SPRY2 expression in human granulosa cells

**Figure 1:**
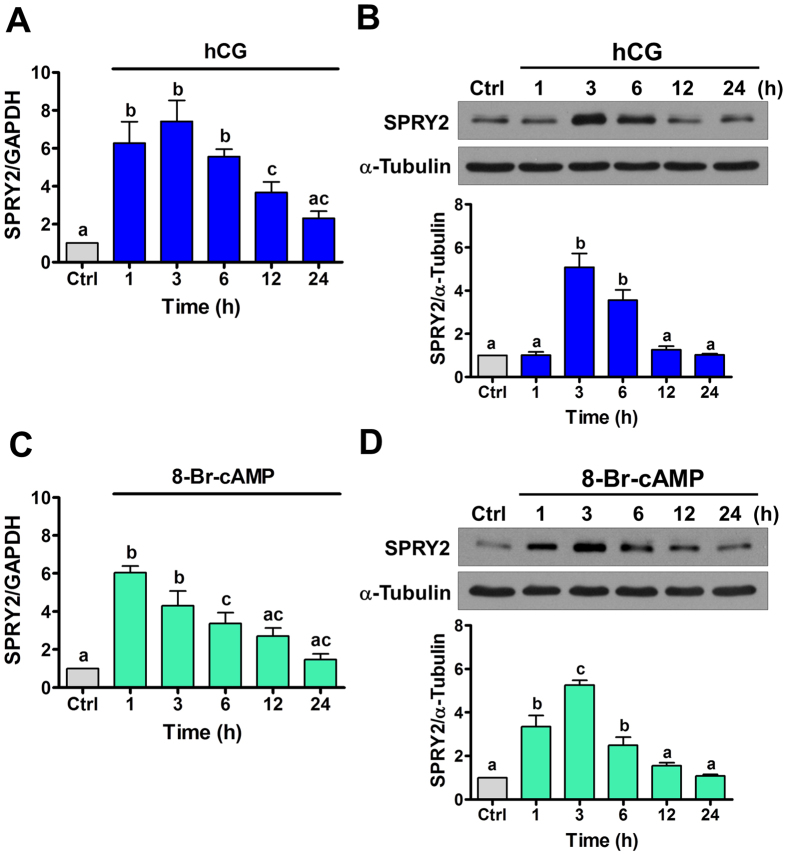
hCG up-regulates SPRY2 expression in SVOG cells. (**A,C**) Cells were treated with vehicle control (Ctrl), 10 IU/mL hCG (**A**) or 1 mM 8-Br-cAMP (**C**) for various periods of time, and the mRNA levels of SPRY2 were examined by RT-qPCR. The level of SPRY2 mRNA at each time point was normalized to that of GAPDH mRNA at the same time point. (**B,D**) Cells were treated with vehicle control (Ctrl), 10 IU/mL hCG (**B**) or 1 mM 8-Br-cAMP (**D**) for various periods of time, and the protein levels of SPRY2 were examined by western blot. The results are expressed as the mean ± SEM of at least three independent experiments. Values without a common letter are significantly different (p < 0.05).

**Figure 2:**
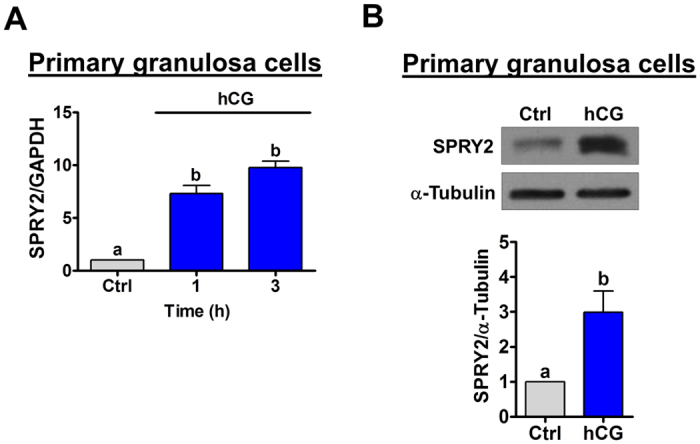
hCG up-regulates SPRY2 expression in primary cultures of human granulosa cells. (**A**) Cells were treated with vehicle control (Ctrl) or 10 IU/mL hCG for 1 and 3 h, and the mRNA levels of SPRY2 were examined by RT-qPCR. The level of COX-2 mRNA at each time point was normalized to that of GAPDH mRNA at the same time point. (**B**) Cells were treated with vehicle control (Ctrl) or 10 IU/mL hCG for 3 h. The protein levels of SPRY2 were examined by western blot. The results are expressed as the mean ± SEM of four independent experiments. Values without a common letter are significantly different (p < 0.05).

SVOG cells are immortalized human granulosa cells obtained from a patient undergoing *in vitro* fertilization that have been shown via examination of the progesterone production to be responsive to hCG^17^. In this study, for technical feasibility, we used this cell model to examine the effect of hCG on SPRY2 expression in human granulosa cells. First, to examine the effect of hCG on SPRY2 expression, SVOG cells were treated with 10 IU/mL hCG for various periods of time. As shown in Fig. 1A, 1 h of treatment with hCG significantly up-regulated SPRY2 mRNA levels. The most significant effect was observed after 3 h of hCG treatment. Western blot results showed that treatment with hCG up-regulated SPRY2 protein levels, with the maximal effect observed 3 h after treatment and the up-regulated SPRY2 protein levels declining after 24 h of hCG treatment (Fig. 1B). To further confirm these results, SVOG cells were treated with 1 mM cell-permeable cAMP analog, 8-Br-cAMP, to mimic the actions of hCG. As shown in Fig. 1C, similar to hCG, treatment with 8-Br-cAMP up-regulated SPRY2 mRNA levels, although the most significant effect was observed after 1 h of 8-Br-cAMP treatment. Moreover, western blot results confirmed the stimulatory effect of 8-Br-cAMP on SPRY2 protein expression (Fig. 1D). We were aware that immortalized granulosa cells might not fully be representative of normal granulosa cells. Therefore, primary cultures of human granulosa cells were treated with hCG to further confirm the effect of hCG on SPRY2 expression. As shown in Fig. 2, similar to SVOG cells, treatment with hCG significantly up-regulated SPRY2 mRNA and protein levels in primary cultures of human granulosa cells.

### ERK1/2 signaling is required in hCG-induced SPRY2 expression in human granulosa cells

**Figure 3:**
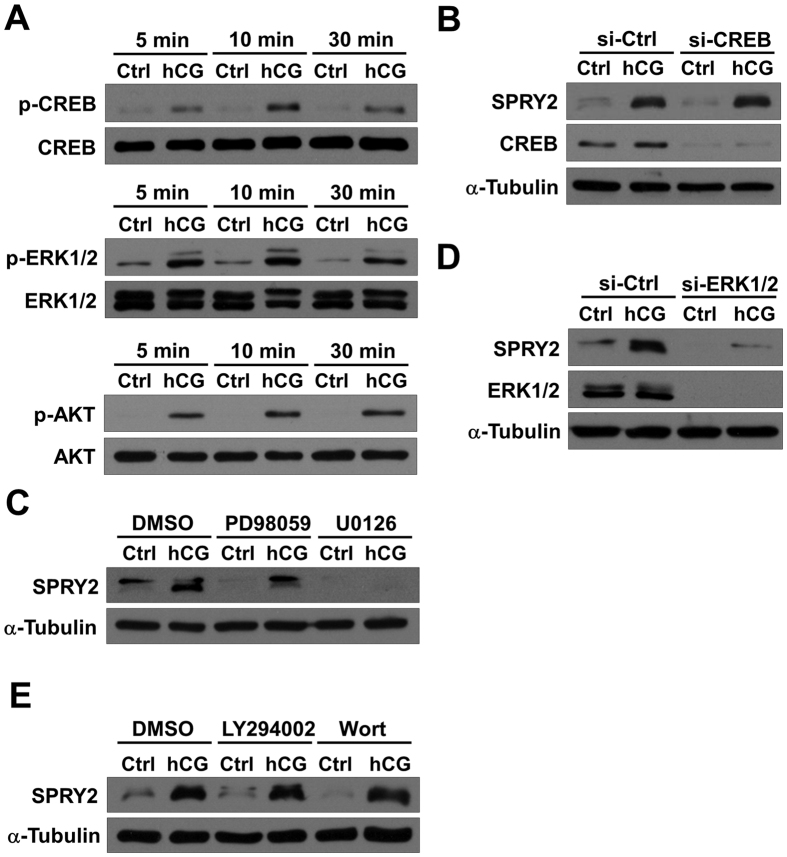
Activation of ERK1/2 signaling is required for hCG-induced up-regulation of SPRY2. (**A**) SVOG cells were treated with vehicle control (Ctrl) or 10 IU/mL hCG for the indicated durations. The phosphorylation levels of CREB, ERK1/2 and AKT were examined by western blot. (**B**) SVOG cells were transfected with 50 nM control siRNA (si-Ctrl) or CREB siRNA (si-CREB) for 48 h and then treated with vehicle control (Ctrl) or 10 IU/mL hCG for 3 h. The protein levels of SPRY2 and CREB were examined by western blot. (**C**) SVOG cells were pre-treated with vehicle control (DMSO), 10 μM PD98059 or 10 μM U0126 for 1 h and then treated with vehicle control (Ctrl) or 10 IU/mL hCG for 3 h. The protein levels of SPRY2 were examined by western blot. (**D**) SVOG cells were transfected with 50 nM control siRNA (si-Ctrl) or ERK1/2 siRNAs (si-ERK1/2) for 48 h and then treated with vehicle control (Ctrl) or 10 IU/mL hCG for 3 h. The protein levels of SPRY2 and ERK1/2 were examined by western blot. (**D**) SVOG cells were pre-treated with vehicle control (DMSO), 10 μM LY294002 or 10 μM Wortmannin (Wort) for 1 h and then treated with vehicle control (Ctrl) or 10 IU/mL hCG for 3 h. The protein levels of SPRY2 were examined by western blot.

LH/hCG has been shown to activate the PKA/CREB, ERK1/2 and PI3K/AKT signaling pathways^18^. As shown in Fig. 3A, treatment with hCG for 5, 10, or 30 min increased the phosphorylation levels of CREB, ERK1/2 and AKT in SVOG cells. To further examine which signaling pathways were involved in the hCG-induced up-regulation of SPRY2 expression, pharmacological inhibitors or specific siRNAs were used to block the activation of signaling pathways that can be activated by hCG. Transfection of cells with CREB siRNA significantly down-regulated the endogenous expression CREB, but knockdown of CREB did not affect the hCG-induced up-regulation of SPRY2 protein levels (Fig. 3B). Interestingly, treatment of cells with a MEK inhibitor, either PD98059 or U0126, attenuated the hCG-induced up-regulation of SPRY2 protein levels (Fig. 3C). To further confirm these results, ERK1/2 siRNAs were used to knock down the endogenous expression of ERK1/2. As shown in Fig. 3D, similar to the results obtained from the inhibitor experiments, knockdown of ERK1/2 attenuated the hCG-induced up-regulation of SPRY2 protein levels. In addition, we used two PI3K inhibitors, LY294002 and Wortmannin, to block activation of the PI3K/AKT signaling pathway. As shown in Fig. 3E, neither LY294002 nor Wortmannin affected the stimulatory effect of hCG on SPRY2 expression. Taken together, these results indicated that ERK1/2 was required for the hCG-induced up-regulation of SPRY2 expression in human granulosa cells.

### SPRY2 knockdown attenuates AREG-induced COX-2 expression and PGE2 production in human granulosa cells

**Figure 4:**
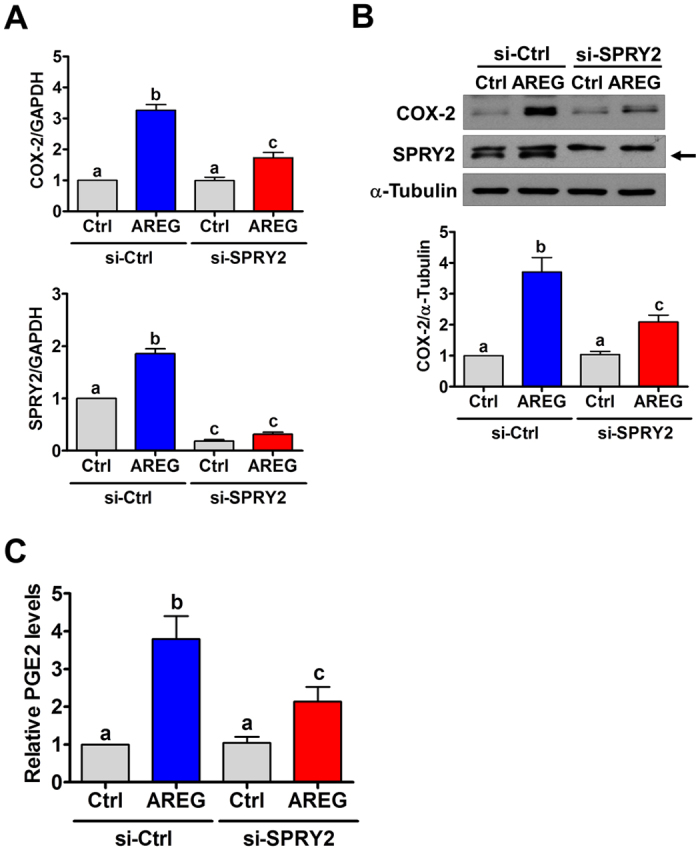
SPRY2 knockdown attenuates AREG-induced COX-2 expression and PGE2 production. (**A,B**) SVOG cells were transfected with 50 nM control siRNA (si-Ctrl) or SPRY2 siRNA (si-SPRY2) for 48 h and then treated with vehicle control (Ctrl) or 100 ng/mL AREG for 1 h. The mRNA (**A**) and protein (**B**) levels of COX-2 and SPRY2 were examined by RT-qPCR and western blot, respectively. The arrow indicates the SPRY2 band. (**C**) SVOG cells were transfected with 50 nM control siRNA (si-Ctrl) or SPRY2 siRNA (si-SPRY2) for 48 h and then treated with vehicle control (Ctrl) or 100 ng/mL AREG for 3 h. The levels of PGE2 in the culture media were examined by ELISA. The results are expressed as the mean ± SEM of at least three independent experiments. Values without a common letter are significantly different (p < 0.05).

We have previously shown that LH/hCG increases AREG expression, which in turn stimulates COX-2 expression in SVOG cells^15^. To examine the role of SPRY2 in AREG-induced COX-2 expression, siRNA-mediated knockdown was used to reduce the endogenous expression of SPRY2. As shown in Fig. 4A, similar to our previous results, treatment with AREG up-regulated COX-2 mRNA levels. Knockdown of SPRY2 did not affect the basal levels of COX-2 mRNA levels, but it attenuated the AREG-induced up-regulation of COX-2 mRNA levels. Similarly, AREG-induced up-regulation of COX-2 protein levels was attenuated by SPRY2 knockdown (Fig. 4B). It has been shown that AREG binds exclusively to EGFR and that EGF can up-regulate SPRY2 expression^19^. Therefore, it is interesting to note that treatment with AREG also up-regulated SPRY2 mRNA and protein levels in SVOG cells (Fig. 4A,B). PGE2, a major COX product, has been well characterized as regulating various reproductive functions in females^20^. Therefore, we also examined the effect of SPRY2 knockdown on AREG-induced PGE2 production. The ELISA results showed that treatment with AREG significantly induced PGE2 production, and this effect was attenuated by knockdown of SPRY2 (Fig. 4C).

### SPRY2 knockdown attenuates AREG-induced activation of ERK1/2 signaling in human granulosa cells

**Figure 5:**
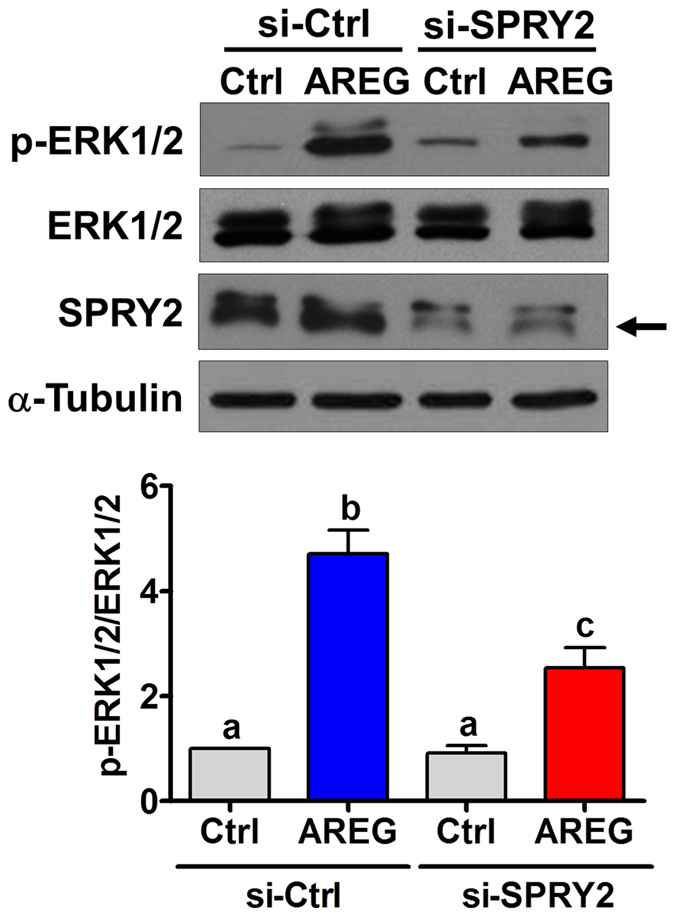
SPRY2 knockdown attenuates AREG-activated ERK1/2 signaling. SVOG cells were transfected with 50 nM control siRNA (si-Ctrl) or SPRY2 siRNA (si-SPRY2) for 48 h and then treated with vehicle control (Ctrl) or 100 ng/mL AREG for 10 min. The levels of ERK1/2 phosphorylation and of SPRY2 expression were examined by western blot. The arrow indicates the SPRY2 band. The results are expressed as the mean ± SEM of at least three independent experiments. Values without a common letter are significantly different (p < 0.05).

Given the inhibitory role of SPRY proteins in regulating the RAS/ERK1/2 signaling pathway, we next examined whether knockdown of SPRY2 affects the AREG-induced activation of ERK1/2 signaling. Consistent with our previous study, treatment with AREG activated ERK1/2 signaling in SVOG cells^15^. In addition, knockdown of SPRY2 attenuated AREG-induced activation of ERK1/2 signaling (Fig. 5). These results indicated that the SPRY2 expression level played an important role in modulating AREG-induced ERK1/2 activation in human granulosa cells.

### SPRY2 expression levels are increased in granulosa cells of patients with ovarian hyperstimulation syndrome

**Figure 6:**
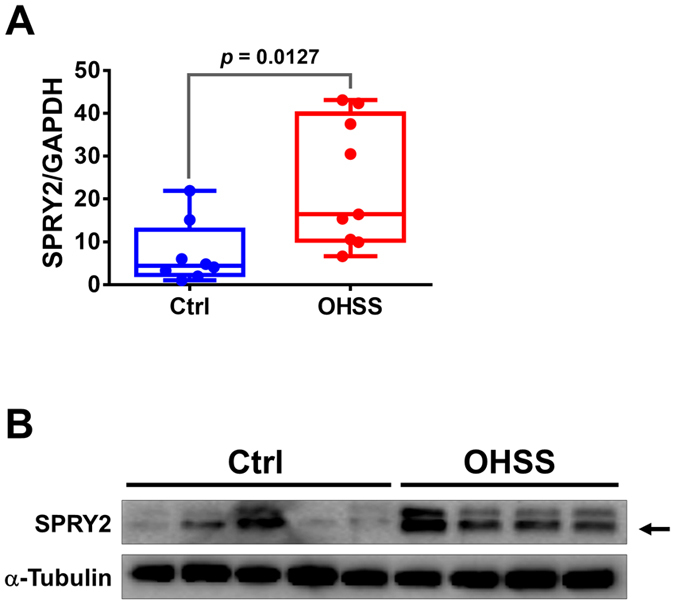
SPRY2 expression levels are increased in granulosa cells of patients with ovarian hyperstimulation syndrome (OHSS). The granulosa cells were obtained from normal IVF patients (N = 8) and IVF patients with high risk of developing OHSS (N = 9). (**A**) The SPRY2 mRNA levels were examined by RT-qPCR. (**B**) The SPRY2 protein levels were examined by western blot. The arrow indicates the SPRY2 band. The RT-qPCR results are expressed as the mean ± SEM.

Ovarian hyperstimulation syndrome (OHSS) is a severe complication of inducing ovulation with hCG. It has been shown that treatment with a COX-2 inhibitor attenuates the main symptoms of this syndrome in an OHSS rat model^21^. Given the role of SPRY2 in the regulation of AREG-induced COX-2 expression and PGE2 production, we were interested in examining the expression levels of SPRY2 in granulosa cells from OHSS patients. As shown in Fig. 6A, compared to the granulosa cells of normal patients, the SPRY2 mRNA levels were significantly increased in the granulosa cells of OHSS patients. Western blot results further confirmed the increase of SPRY2 protein levels in granulosa cells of OHSS patients (Fig. 6B). These results strongly suggested that aberrant expression of SPRY2 may contribute to the pathogenesis of OHSS.

## Discussion

As with all proteins, the level of SPRY2 protein in the cells is determined by the balance between protein synthesis and degradation. The transcriptional regulation of SPRY2 in response to growth factor stimulation was one of the first mechanisms revealed to regulate SPRY2 expression. It has been shown that EGF, fibroblast growth factor (FGF) or platelet-derived growth factor (PDGF) up-regulates SPRY2 expression by activating the ERK1/2 signaling pathway^19^. Although MAPK signaling has been considered the major signaling pathway to stimulate SPRY2 expression, a study in mouse chondrogenic cells shows that calcium-dependent signaling and the PLCγ signaling pathway are also involved in regulating SPRY2 expression^22^. To the best of our knowledge, thus far, only a handful of studies have examined the regulation of SPRY2 in the ovary^7^,^16^,^23^,^24^. In mouse cumulus cells, FGF1, FGF2, FGF8 and EGF are able to stimulate SPRY2 expression^23^. In addition, the ERK1/2, AKT and calcium-dependent signaling pathways are involved in the FGF2-induced up-regulation of SPRY2 expression in bovine granulosa cells^24^. Although FGF and EGF have been shown to increase the expression of SPRY2 in human granulosa cells, the underlying molecular mechanisms remain to be characterized^7^. In the present study, consistent with the previous finding^16^, our results showed that treatment with hCG up-regulated SPRY2 expression in human granulosa cells. In addition, by using pharmacological inhibitors and siRNA-mediated knockdown, we showed that activation of ERK1/2 was required for the hCG-induced up-regulation of SPRY2 expression in human granulosa cells. Binding sites for several transcription factors, including SP1, AP2, CREB and Ets-1, have been identified on the human SPRY2 promoter, and these transcription factors may play important roles in the regulation of SPRY2 expression^25^. Although hCG activated CREB in human granulosa cells, knockdown of CREB did not affect the hCG-induced up-regulation of SPRY2. These results indicated that CREB is not required for the hCG-induced expression of SPRY2 in human granulosa cells. Therefore, future studies will be needed to examine the detail transcriptional machinery that mediates the hCG-induced SPRY2 expression in human granulosa cells.

Animal studies and our previous study have demonstrated the stimulatory role of EGF-like growth factors, AREG, BTC and EREG, in the regulation of COX-2 expression and PGE2 production in granulosa cells^12^,^15^. These results indicate that tight modulation of EGFR-mediated signaling is important to ensure that the ovary functions normally. Our previous study shows that treatment of SVOG cells with LH/hCG for 1 h significantly increases the expression levels of AREG and EREG, although significant effects of BTC induction are observed after 6 h of treatment^15^. Interestingly, in the present study, SPRY2 protein levels were up-regulated by 3 h of hCG treatment. Therefore, although we do not have any direct evidence to exclude the effect of SPRY2 in hCG-induced ERK1/2 signaling, the time frames for these pathways suggested that hCG-induced up-regulation of SPRY2 mainly affects the signaling pathways activated by EGF-like growth factors. Indeed, our results showed that knockdown of SPRY2 attenuated AREG-activated ERK1/2 signaling. These results indicate that hCG-induced SPRY2 plays a critical role to modulate the effects of EGFR-mediated cellular signaling pathways.

SPRY2 has been shown to be expressed in human granulosa cells^7^. However, the function of SPRY2 in human granulosa cells remains largely unknown and must be defined. The regulation of EGFR signaling by SPRY2 is more complicated than that of FGFR because SPRY2 can function as a positive or a negative regulator of EGFR-mediated MAPK signaling in a domain-dependent fashion^26^. Ectopic expression of full-length human SPRY2 results in a potentiation of EGFR-mediated MAPK activation. In contrast, ectopic expression of the human SPRY2 C-terminal cysteine-rich domain attenuates EGF-induced MAPK activation^26^. It has been shown that EGF stimulation induces the binding between SPRY2 and the E3 ubiquitin ligase c-Cbl, which promotes the ubiquitylation and proteolytic degradation of SPRY2 protein^27^,^28^,^29^. Interestingly, the formation of a complex between SPRY2 and c-Cbl also affects the EGF/EGFR-induced signaling because c-Cbl normally binds to the activated EGFR and promotes its ubiquitylation and degradation^30^. Therefore, SPRY2 can prevent the degradation of activated EGFR by competing for binding with c-Cbl, thereby prolonging the EGF-mediated signaling^26^,^31^. AREG has been shown to bind exclusively to EGFR^32^. Our recent study shows that AREG treatment induces EGFR down-regulation in human granulosa cells^33^. In the present study, AREG-induced activation of ERK1/2 signaling was significantly attenuated by knockdown of SPRY2. Taken together, these results clearly indicate that SPRY2 can sustain AREG-activated signaling by preventing the down-regulation of EGFR in human granulosa cells.

Ovarian hyperstimulation syndrome (OHSS) is a serious iatrogenic complication in women undergoing induction of ovulation with hCG for assisted reproductive techniques. The incidence of moderate OHSS is 3–6%, and the severe form may occur in 0.1–2% of IVF cycles. The incidence may reach as high as 20% in the high-risk group. The main characteristics of OHSS are cystic ovarian enlargement, increased vascular permeability and extravasation of fluid from intravascular to extravascular space^34^,^35^,^36^. Interestingly, a few recent studies in rat OHSS models have shown that COX-2 expression is significantly increased in OHSS ovary and that inhibition of COX-2 can suppress the occurrence of OHSS^21^,^37^,^38^. We have previously demonstrated that AREG induces COX-2 expression in human granulosa cells by activating EGFR-mediated ERK1/2 signaling^15^. In the present study, our results demonstrated for the first time that the expression of SPRY2 was significantly increased in the granulosa cells of OHSS patients. These novel clinical results, combined with the positive regulatory role of SPRY2 on the EGFR-mediated ERK1/2 signaling, suggest that after the LH surge, EGF-like growth factors, such as AREG, BTC and EREG, produced by granulosa cells activate EGFR to induce COX-2 expression and PGE2 production through an autocrine/paracrine mechanism. However, activated EGFR may be rapidly down-regulated by protein degradation, which would negatively regulate EGFR-mediated cellular functions^39^. In OHSS patients, the higher expression levels of SPRY2 in granulosa cells may prevent the degradation of activated EGFR, leading to prolonged signaling. Therefore, in this context, the AREG-induced COX-2 expression may be enhanced, resulting in the overexpression of COX-2 in the OHSS ovary. Taken together, our results indicate that aberrant expression of SPRY2 may contribute to the pathogenesis of OHSS.

In summary, the present study demonstrates that the expression of SPRY2 is up-regulated by hCG treatment in human granulosa cells. In addition, the ERK1/2 signaling pathway is required for hCG-induced up-regulation of SPRY2 expression. Knockdown of SPRY2 attenuates the levels of AREG-induced COX-2 expression and PGE2 production by inhibiting the AREG-induced activation of the ERK1/2 signaling pathway. Moreover, our results demonstrate that SPRY2 expression levels are significantly higher in granulosa cells of OHSS patients than in granulosa cells of non-OHSS patients. These results elucidate the physiological roles of SPRY2 in granulosa cells and provide important insight into the molecular mechanisms that mediate the pathogenesis of OHSS.

## Materials and Methods

### Cell culture

A non-tumorigenic immortalized human granulosa cell line (SVOG) that was previously established by our group was used. This cell line was produced by the SV40 large T antigen transfection of early luteal phase human granulosa cells obtained from women undergoing *in vitro* fertilization^17^. Cells were grown in DMEM/F12 medium (Sigma, Oakville, ON) supplemented with 10% charcoal/dextran-treated fetal bovine serum (Hyclone Laboratories Inc., Logan, UT). The cultures were maintained at 37 °C in a humidified atmosphere of 5% CO_2_.

### Preparation of primary human granulosa cells

Primary human granulosa cells were obtained with informed patient consent following approval from the Zhengzhou University Research Ethics Board. The study was carried out in accordance with the approved guidelines from the Zhengzhou University Research Ethics Board. The controlled ovarian stimulation protocol for *in vitro* fertilization patients consisted of either luteal-phase nafarelin acetate or the follicular-phase GnRH antagonist down-regulation. Gonadotropin stimulation began on menstrual cycle day 2 with human menopausal gonadotropin and recombinant FSH (Gonal-F; Merck, Darmstadt, Germany) and was followed by human chorionic gonadotropin administration 34–36 h before oocyte retrieval, which was based on follicle size. Granulosa cells were purified by density centrifugation from follicular aspirates collected from women undergoing oocyte retrieval as previously described^40^. The criteria for considering a patient at risk of developing ovarian hyperstimulation syndrome (OHSS) were a serum estradiol level >3000 pg/mL on the day of hCG administration and the retrieval of >20 oocytes^41^.

### Antibodies and reagents

Polyclonal anti-Sprouty2 antibody was obtained from Sigma. The monoclonal anti-phospho-ERK1/2 (Thr202/Tyr204) and polyclonal anti-phospho-CREB, anti-CREB, anti-ERK1/2, anti-phospho-AKT (Ser473) and anti-AKT antibodies were obtained from Cell Signaling (Danvers, MA). The polyclonal anti-COX-2 antibody was obtained from Abcam (Cambridge, MA). The monoclonal anti-α-tubulin antibody was obtained from Santa Cruz Biotechnology (Santa Cruz, CA). Horseradish peroxidase-conjugated goat anti-mouse and goat anti-rabbit IgG were obtained from Bio-Rad Laboratories (Hercules, CA). Human chorionic gonadotropin (hCG), 8-bromoadenosine 3′,5′-cyclic monophosphate (8-Br-cAMP) and LY294002 were obtained from Sigma. Recombinant human amphiregulin was obtained from R&D systems (Minneapolis, MN). Wortmannin, PD98059 and U0126 were obtained from Calbiochem (San Diego, CA).

### Reverse transcription quantitative real-time PCR (RT-qPCR)

Total RNA was extracted using TRIzol reagent (Invitrogen, Life Technologies, Burlington, ON) in accordance with the manufacturer’s instructions. Reverse transcription was performed with 3 μg of RNA, random primers and M-MLV reverse transcriptase (Promega, Madison, WI). The primers were designed using Primer Express Software v2.0 (Applied Biosystems, Foster City, CA). All primers spanned at least one intron in order to detect specific mRNA sequences. The following primers were used for SYBR Green reverse transcription-qPCR (RT-qPCR): Sprouty2: 5′-TGG CAA GTG CAA ATG TAA GG-3′ (sense) and 5′-ACC ATC GTG TAC AAC AGT GA-3′ (antisense); COX-2: 5′-CCC TTG GGT GTC AAA GGT AA-3′ (sense) and 5′-GCC CTC GCT TAT GAT CTG TC-3′ (antisense) and GAPDH: 5′-GAG TCA ACG GAT TTG GTC GT-3′ (sense) and 5′-GAC AAG CTT CCC GTT CTC AG-3′ (antisense). RT-qPCR was performed using the Applied Biosystems 7300 Real-Time PCR System equipped with a 96-well optical reaction plate. The specificity of each assay was validated by melting curve analysis and agarose gel electrophoresis of PCR products. All of the RT-qPCR experiments were run in triplicate, and a mean value was used to determine the mRNA levels. Water and mRNA without RT were used as negative controls. Relative quantification of the mRNA levels was performed using the comparative Ct method with GAPDH as the reference gene and the formula 2^−∆∆Ct^.

### Western blot

Cells were lysed in cell lysis buffer (Cell Signaling). Equal amounts of protein were separated by SDS polyacrylamide gel electrophoresis and transferred onto PVDF membranes. After 1 h of blocking with 5% non-fat dry milk in Tris-buffered saline (TBS), the membranes were incubated overnight at 4 °C with primary antibodies that were diluted in 5% non-fat milk-TBS. Following primary antibody incubation, the membranes were incubated with the appropriate HRP-conjugated secondary antibody. Immunoreactive bands were detected using an enhanced chemiluminescent substrate and X-ray film. The intensities of bands were quantified by densitometric analysis using Scion Image software (Scion Corp, Frederick, MD, USA).

### Small interfering RNA (siRNA) transfection

To knock down endogenous CREB, ERK1/2 or Sprouty2, cells were transfected with 50 nM ON-TARGET*plus* SMART*pool* CREB, ERK1, ERK2 or Sprouty2 siRNA (Dharmacon, Lafayette, CO) using Lipofectamine RNAiMAX (Invitrogen, Life Technologies). siCONTROL NON-TARGETING *pool* siRNA (Dharmacon) was used as the transfection control.

### Prostaglandin E2 ELISA

A human PGE2-specific ELISA was used in accordance with the manufacturer’s protocol (Cayman Chemical, Ann Arbor, MI). The culture media were collected, and the PGE2 levels in the culture media were measured by ELISA. PGE2 levels were normalized to the protein concentrations from the cell lysates. Normalized PGE2 values from the treatments are represented as relative values in comparison to the control treatment.

### Statistical analysis

The results are presented as the mean ± SEM of at least three independent experiments. GraphPad Prism software was used for statistical analysis. Multiple comparisons were analyzed by one-way ANOVA followed by Tukey’s multiple comparison tests. A significant difference was defined as *p* < 0.05.

## Additional Information

**How to cite this article**: Cheng, J.-C. *et al*. hCG-induced Sprouty2 mediates amphiregulin-stimulated COX-2/PGE2 up-regulation in human granulosa cells: a potential mechanism for the OHSS. *Sci. Rep.* **6**, 31675; doi: 10.1038/srep31675 (2016).
